# Construction of a Novel LncRNA Signature Related to Genomic Instability to Predict the Prognosis and Immune Activity of Patients With Hepatocellular Carcinoma

**DOI:** 10.3389/fimmu.2022.856186

**Published:** 2022-04-08

**Authors:** Jinfeng Zhu, Qian Huang, Sicheng Liu, Xingyu Peng, Ju Xue, Tangbin Feng, Wulang Huang, Zhimeng Chen, Kuiyuan Lai, Yufei Ji, Miaomiao Wang, Rongfa Yuan

**Affiliations:** ^1^ Department of General Surgery, The Second Affiliated Hospital of Nanchang University, Nanchang, China; ^2^ Jiangxi Province Key Laboratory of Molecular Medicine, The Second Affiliated Hospital of Nanchang University, Nanchang, China; ^3^ Department of General Practice, The Third Xiangya Hospital, Central South University, Changsha, China; ^4^ Department of Pathology, Hunan Provincial People’s Hospital, The First Affiliated Hospital of Hunan Normal University, Changsha, China; ^5^ Department of Surgery, II, Duchang County Hospital of Traditional Chinese Medicine, Jiujiang, China; ^6^ Department of General Surgery, Affiliated Hospital of Jinggangshan University, Jian, China; ^7^ The Second Clinical Medical College of Nanchang University, Nanchang, China; ^8^ Queen Mary College of Nanchang University, Nanchang, China

**Keywords:** genomic instability, hepatocellular carcinoma, prognosis, long non-coding RNAs, signature, immune infiltration, tumor immune activity

## Abstract

**Background:**

Genomic instability (GI) plays a crucial role in the development of various cancers including hepatocellular carcinoma. Hence, it is meaningful for us to use long non-coding RNAs related to genomic instability to construct a prognostic signature for patients with HCC.

**Methods:**

Combining the lncRNA expression profiles and somatic mutation profiles in The Cancer Genome Atlas database, we identified GI-related lncRNAs (GILncRNAs) and obtained the prognosis-related GILncRNAs through univariate regression analysis. These lncRNAs obtained risk coefficients through multivariate regression analysis for constructing GI-associated lncRNA signature (GILncSig). ROC curves were used to evaluate signature performance. The International Cancer Genomics Consortium (ICGC) cohort, and *in vitro* experiments were used for signature external validation. Immunotherapy efficacy, tumor microenvironments, the half-maximal inhibitory concentration (IC50), and immune infiltration were compared between the high- and low-risk groups with TIDE, ESTIMATE, pRRophetic, and ssGSEA program.

**Results:**

Five GILncRNAs were used to construct a GILncSig. It was confirmed that the GILncSig has good prognostic evaluation performance for patients with HCC by drawing a time-dependent ROC curve. Patients were divided into high- and low-risk groups according to the GILncSig risk score. The prognosis of the low-risk group was significantly better than that of the high-risk group. Independent prognostic analysis showed that the GILncSig could independently predict the prognosis of patients with HCC. In addition, the GILncSig was correlated with the mutation rate of the HCC genome, indicating that it has the potential to measure the degree of genome instability. In GILncSig, LUCAT1 with the highest risk factor was further validated as a risk factor for HCC *in vitro*. The ESTIMATE analysis showed a significant difference in stromal scores and ESTIMATE scores between the two groups. Multiple immune checkpoints had higher expression levels in the high-risk group. The ssGSEA results showed higher levels of tumor-antagonizing immune cells in the low-risk group compared with the high-risk group. Finally, the GILncSig score was associated with chemotherapeutic drug sensitivity and immunotherapy efficacy of patients with HCC.

**Conclusion:**

Our research indicates that GILncSig can be used for prognostic evaluation of patients with HCC and provide new insights for clinical decision-making and potential therapeutic strategies.

## Introduction

Liver cancer is the third leading cause of cancer-related death globally (1). There are approximately 850,000 new cases each year, of which hepatocellular carcinoma (HCC) accounts for approximately 90% of all primary liver cancer cases (2). HCC is associated with hepatitis B virus (HBV) and hepatitis C virus infection, cirrhosis, alcoholism, and non-alcoholic fatty liver (3). These factors directly or indirectly damage the DNA, thereby generating mutations and promoting genome instability (4–7). Genomic instability (GI) refers to various DNA changes, from changes in a single nucleotide to changes in an entire chromosome (8). Studies have reported that GI is a characteristic of most cancer types and is considered as the driving force of cancer development (9). Generally, GI promotes an increase in the genetic changes characteristic of cancer, which in turn promotes cancer progression (8). For example, HBV DNA integration promotes local and distant oncogenic driving changes in HCC (10). Zhou Y et al. have reported that NOD2 activates the nuclear autophagy pathway during the occurrence of liver cancer, leading to impaired DNA damage repair and increased GI (11). Therefore, the identification of biomarkers related to GI is of great significance for the early diagnosis and prognostic evaluation of patients with HCC.

With in-depth research on GI, researchers have found that GI determines the behavior of cancer cells and their response to treatment (12). Features of genomic instability have been reported to be associated with response to immune-directed therapy (13). In addition, recent studies have revealed that innate immunity induced by DNA damage can be used as a new target for cancer treatment, which has expanded the scope of immunotherapy (14). However, research in this field of GI and immunotherapy of HCC is still limited.

New research has pointed out that long non-coding RNAs (lncRNAs) are involved in the induction of GI (15–17). LncRNAs are non-protein-coding RNA molecules (18) of more than 200 nucleotides. Due to their location and specific interactions with the DNA, RNA, and proteins, lncRNAs have multiple functions. For example, they modulate chromatin function, regulate the assembly and function of membrane-less nuclear bodies, alter the stability and translation of cytoplasmic mRNAs, and interfere with signaling pathways (19). At the same time, lncRNA monitoring also plays an important role in coordinating various biological processes, including the protection of genome integrity, diversification of antibody genes, regulation of heterochromatin formation, coordination of immune cell activation and maintenance of the pluripotency of embryonic stem cells, among others (20, 21). With the in-depth exploration of the function of lncRNAs, researchers have found that it is a resident staff of GI regulation in tumorigenesis (22). For example, the study by Munschauer M et al. (23) showed that noncoding RNAs activated by DNA damage (NORAD) were involved in genome stability. In addition, the study by Hu WL et al. (24) demonstrated that p53-responsive lncRNA (GUARDIN) was important for maintaining genome integrity under steady-state conditions and after exposure to exogenous genotoxic stress. Furthermore, the work by Tracy K and colleagues (25) showed that mitosis-related lncRNA (MANCR) affected the genome stability and cell division of invasive breast cancer.

Therefore, it is necessary to explore the lncRNAs related to GI for the early diagnosis and prognostic evaluation of HCC.

## Materials and Methods

### Data Collection and Processing

First of all, the gene transcriptome data (n = 424), gene mutation data (n = 364) and clinical data (n = 377) of patients with HCC were downloaded from the TCGA database (https://portal.gdc.cancer.gov/). Fragments per Kilobase million was used for the transcriptome data, and the VarScan version was used for the gene mutation data. Then, we sorted out the gene expression levels of all lncRNAs from the transcriptome data (normal: 50; tumor: 374) and eliminated the normal samples. Next, we collected survival information for HCC patients from clinical data, excluding samples with incomplete survival information (n = 1) and survival time < 30 days (n = 27) to reduce the interference of non-cancer deaths. After matching the data that met the above conditions, we finally obtained 343 cases with the corresponding gene transcriptome data and clinical data.

### Screening of the GILncRNAs

Consistent with previous studies (26), we assigned the top 25% and bottom 25% samples in descending order of number of mutations to the Genomically Unstable (GU) and Genomically Stable (GS) groups, separately. Then, the IDs of the samples of the two groups were matched with the LncRNA expression data to obtain the LncRNA expression levels of the GU and GS groups. Finally, Wilcoxon rank-sum test was used on the gene expression levels of the GU and GS groups, and the lncRNAs with a log fold change (logFC) > 1 and a false discovery rate (FDR) < 0.05 were defined as GILncRNAs.

To verify the correlation between the GILncRNAs and GI, we extracted the LncRNA expression data of all tumor samples (n = 374) and conducted a hierarchical cluster analysis of them by calculating the Euclidean distances and cutting the tree into two clusters. We defined one which had a high number of mutations as genomic unstable (GU-like) cluster and the other that had a low number of mutations as genomic stable (GS-like) cluster. Next, we verified the differences between the GU-like and GS-like clusters regarding the number of somatic mutations and the *NOD2 (* 11) gene expression levels using the Mann-Whitney U test.

### Identification of a Genomic Instability-Related lncRNA Signature

The GILncRNA samples matched with the survival data and expression data were randomly and equally allocated to the training and testing sets. We performed univariate Cox regression analysis on GILncRNAs in the training set and identified GILncRNAs associated with prognosis. Then these GIlncRNAs were analyzed by multiple Cox regression to determine their respective regression coefficients, and a GILncSig was constructed. The formula of the GILncSig was the following:

$Riskscore=\sum_{i=1}^{n}\exp\left(Xi\right)\times coef\left(Xi\right)$ , where “exp(X_i_)”, “coef(X_i_),” and “n” represented the expression level, the coefficient, and the GILncSig number, respectively.

Furthermore, we calculated the risk scores of all the samples according to the above formula. The samples with higher or lower average risk scores were assigned to high- and low-risk groups, respectively.

### Evaluation and Verification of GILncSig

First, we compared the overall survival (OS) and median OS of patients with HCC in the high- and low-risk groups using the log-rank test. We also drew the ROC curves of the 1-, 3-, and 5-year survival rates of the patients with HCC of the testing set and the TCGA set through time-dependent ROC analysis.

Then, we verified the association of the two risk groups with the number of somatic mutations and NOD2 expression levels using the Mann-Whitney U test. Since the genes had wild and mutant states, we analyzed the proportion of the *TP53* mutation states between the high- and the low-risk groups using a Chi-squared test. We also implemented a joint survival analysis based on the *TP53* mutation status and two clusters using the log-rank test.

Next, we completed univariate and multivariate Cox regression analyses of the risk score and other clinical factors to verify the independence of GILncSig.

Finally, we compared the area under the ROC curve of GILncSig with others for predicting OS at 1-, 2-, and 3-year.

RNAseq data (grade 3) and corresponding clinical information were obtained from the ICGC database (https://dcc.icgc.org/releases/current/Projects) for 240 cases of primary liver cancer (LIRI-JP). Log-rank was used to test the survival difference between the high and low gene expression groups in the KM survival analysis.

### Samples

A total of 37 cancer and paracancerous tissues diagnosed with HCC were collected from the Second Affiliated Hospital of Nanchang University. This study passed the review of the Medical Ethics Committee of the Second Affiliated Hospital of Nanchang University. The patients included in this experiment were informed and obtained written consent, and this study complied with the standards set by the Declaration of Helsinki.

### Cell Culture and Transfection

The human HCC cell line (MHCC97H) was purchased from the Shanghai Institute of Cell Research, Chinese Academy of Sciences, and the cells were validated by the cell bank of short tandem repeats. MHCC97H was cultured in DMEM medium with 10% fetal bovine serum and cultured at 37°C with 5% carbon dioxide. LUCAT1 interfering fragment siRNA and negative control si-NC were obtained from GenePharma (GenePharma Co., Ltd., Shanghai, China). Cell transfection experiments were performed according to the instructions for using Lipofectamine 3000 Transfection Reagent (Invitrogen, Waltham, Massachusetts, USA). The sequences of siRNA are shown in **Table S1**.

### Quantitative Real-Time PCR

Total RNA was extracted from tissues and cells using the Trizol method, respectively, and then reverse transcribed into cDNA (TaKaRa, RR047A) and used for real-time quantitative PCR (TAKARA, RR420A). Data analysis was performed using the 2^-ΔΔCt^ method. The primer sequences used are listed in **Table S1**.

### Cell Counting Kit-8 (CCK-8) Assay and EdU Assay

The proliferative capacity of MHCC97H with/without LUCAT1 downregulation was observed by CCK-8 assay and EdU assay. Two group cells were seeded in 96-well plates at 6 × 10^3^ cells per well and allowed to adhere. CCK-8 cell proliferation reagent (10 μl) was added to each well at 0h, 24h, 48h, and 72h. 2 hours after CCK-8 administration, the cell proliferation ability was detected with absorbance detection at 450 nm. In addition, two groups of cells were cultured in a 50 μM EdU medium (Guangzhou Ribobio Co., Ltd.) for 2 h. The medium was then discarded and cells were fixed with 4% paraformaldehyde and 0.5% TritonX-100 in PBS to increase cell membrane permeability. Incubate cells with 1 × Apollo Stain Reaction Solution for 30 minutes at room temperature in the dark. Finally, nuclear DNA was stained with 1 × Hoechst33342 for 30 minutes in the dark. Subsequently, the cells were observed under a fluorescence microscope and photographed.

### Transwell Assay and Wound-Healing Assay

MHCC97H cells with/without LUCAT1 downregulation were seeded in transwell chambers at 4 × 104 cells per group. Serum-free medium was used for the upper layer of the chamber, and a complete medium with 15% FBS was used for the lower layer of the chamber. After 24 hours of culture, cells in the lower chamber were fixed with 4% formaldehyde solution and stained with 0.2% crystal violet solution for 20 min. Finally, the cells in the upper layer of the chamber were wiped off with a cotton swab, and the cells migrated to the lower layer of the chamber were observed and counted by an inverted microscope. At the same time, cell migration ability was examined using a wound-healing assay. Briefly, two groups of cells were seeded in 6-well plates and scratched with a sterile pipette (200 μL) when the cells were about 90% confluent. The wound healing was then observed with an inverted microscope at 0 hours, 24 hours, and 48 hours, and photographs were taken.

### Protein Extraction and Western Blot

As previously described (27). Total protein from MHCC97H cells transfected with or without si-LUCAT1 was extracted, and western blotting was performed using the following primary antibodies: anti-PDL1/CD274(1:2000, Proteintech, Wuhan, China) and anti-GAPDH(1:10000, Proteintech, Wuhan, China).

### Gene Set Enrichment Analysis (GSEA)

The top 10 target genes (mRNAs) that were the most related to GILncRNA were obtained by computing the Pearson correlation coefficients, and the lncRNA-mRNA co-expression network was plotted using the Cytoscape software. We used GSEA (28) to look for the functions and signaling pathways associated with GILncSig. For each analysis, gene set permutations were implemented 1,000 times. A *p*-value < 0.05 was regarded as the cut-off criteria for Gene Ontology (GO) and Kyoto Encyclopedia of Genes and Genomes (KEGG) pathway enrichment sorting in GSEA.

### Assessing the Tumor Microenvironment, Immune Checkpoints and Immune Cell Infiltration

The proportions of components in the tumor microenvironment (TME) were assessed using the ESTIMATE algorithm of the “estimate” package (29), resulting in three scores: immune score, stromal score, and ESTIMATE score (30). The higher the score, the greater the proportion of the corresponding component in the TME. We identified potential immune checkpoint genes based on previously published literature (31, 32). The infiltration levels of immune cell types were quantified by ssGSEA in R package gsva (33, 34). ssGSEA is a popular enrichment algorithm, which was extensively utilized in medical studies (35, 36).

### Evaluation of Chemotherapeutic Drug Sensitivity and Immunotherapy Efficacy

To predict chemotherapeutic sensitivity, R-pack “pRRophetic” was used to measure the 50% maximum inhibitory concentration (IC50) of different groups of samples by ridge regression. The IC50 of different groups was then compared by Wilcoxon sign-rank test. Second, immunotherapy responsiveness was predicted using the Tumor Immune Dysfunction and Exclusion (TIDE) Tool (http://tide.dfci.harvard.edu/). As previously shown (37), the GSE78220 dataset was used to analyze efficacy among signature genes and response to immune checkpoint inhibitor treatment.

### Statistical Analysis

Wilcoxon rank-sum test was used for comparing the gene expression levels between the GU and GS groups, as well as different tissue samples. Mann-Whitney U test was used to compare the number of somatic mutations and the *NOD2* gene expression levels between the two groups. A Chi-square test was used to analyze the clinicopathological characteristics between the training and the testing sets. Log-rank test was used for comparing the OS and median OS. Pearson’s correlation test was used to assess the correlation between GILncSig and immune checkpoint expression levels. *p* < 0.05 represented a statistical difference.

## Results

### Filtering the Genomic Instability-Related LncRNAs

**Figure 1:**
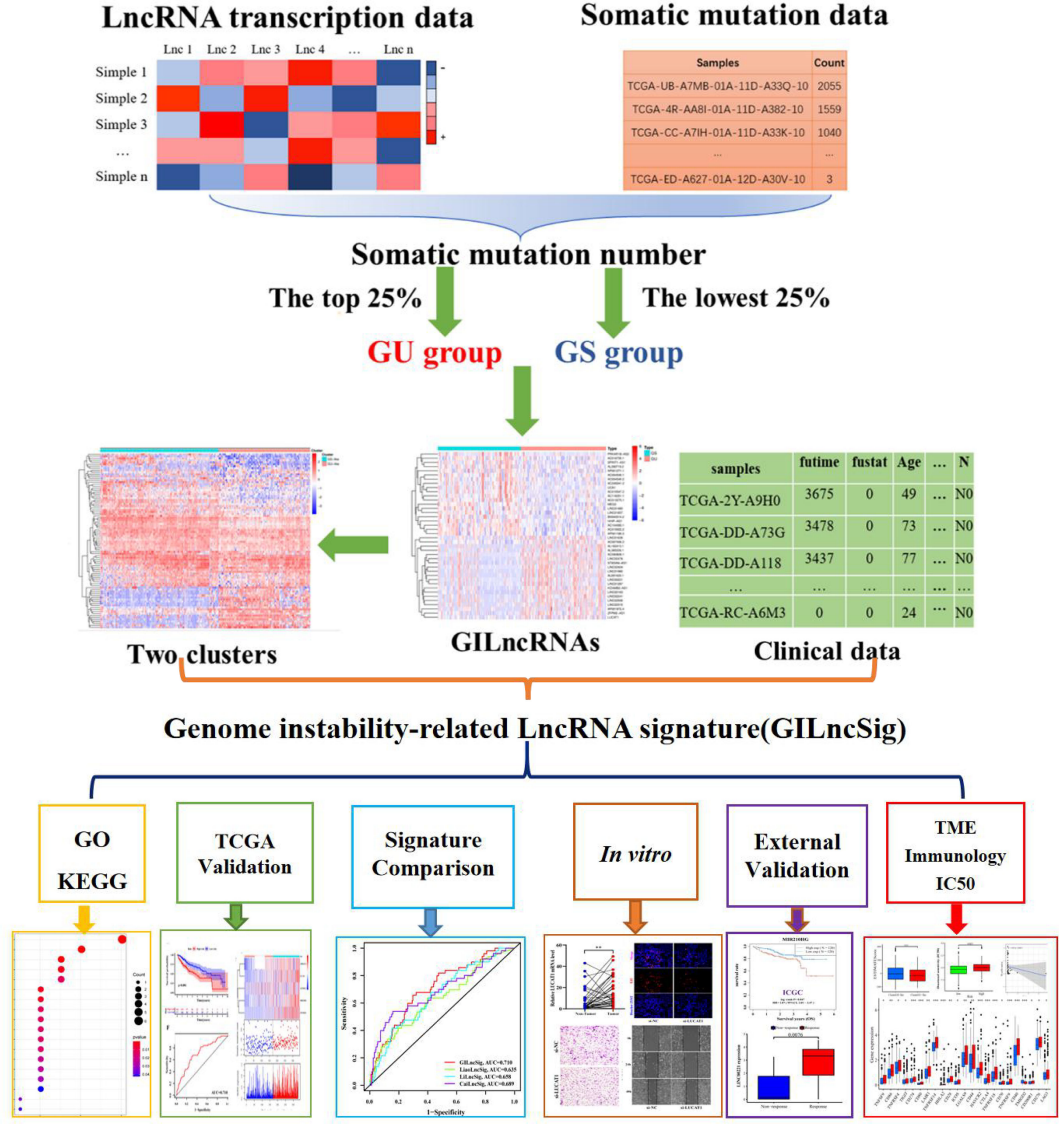
Flow Chart of This Study.

**Figure 2:**
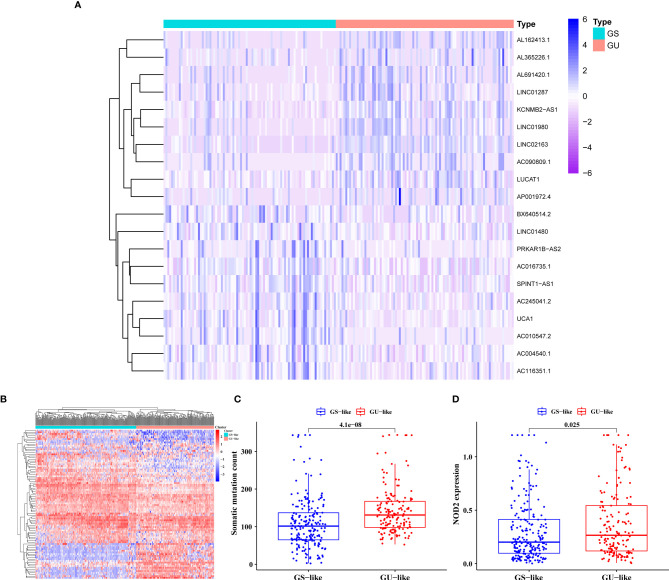
Screening and Validation of the GILncRNAs. **(A)** Genome instability-related lncRNAs (GILncRNAs) were filtered out through differential analysis of the expression levels between the genomic unstable (GU) and the genomic stable (GS) groups. Among them, 10 GILncRNAs with the most obvious up-regulation and 10 GILncRNAs with the most obvious down-regulation were presented in the form of heat maps. **(B)** All GILncRNA samples were divided into two clusters using the clustering algorithm: one containing the samples with a large number of gene mutations, the genomic unstable-like (GU-like) cluster, and another containing the samples with a small number of gene mutations, the genomic stable-like (GS-like) cluster. **(C)** Comparison of the somatic mutations in the GU-like and GS-like clusters. **(D)** Comparison of the *NOD2* expression level in the GU-like and GS-like clusters.

The general process of this study is shown in **Figure 1**. We collated the somatic mutation frequencies for each patient and arranged them in descending order. The patients with the highest and lowest 25% mutation frequencies were then assigned to GU (n = 93) and GS (n = 90) groups, respectively (26). Finally, 88 out of 1,362 lncRNAs were screened and identified as genomic instability-related lncRNAs (GILncRNAs) through the analysis of the gene expression differences between the GU and GS groups (**Table S2**), which was consistent with previous studies (38). In the GU group, 56 genes showed an increased expression and 32 showed a decreased expression. The 10 most upregulated GILncRNAs and the 10 most downregulated GILncRNAs were observed in the heat maps (**Figure 2A**).

We conducted relevant validation to confirm the correlation between these 88 lncRNAs and GI. In the beginning, we classified all patients into the genomic stable-like (GS-like, n = 163) and genomic unstable-like (GU-like, n = 211) clusters (**Figure 2B**) by unsupervised cluster typing. Then, we found significantly higher somatic mutation frequency and NOD2 (11) gene expression in GU-like clusters compared with GS-like clusters (all *p* < 0.05, **Figures 2C, D**). These results confirmed that the lncRNAs we filtered were correlated with GI.

### Constructing a Genomic Instability-Related LncRNA Signature

**Figure 3:**
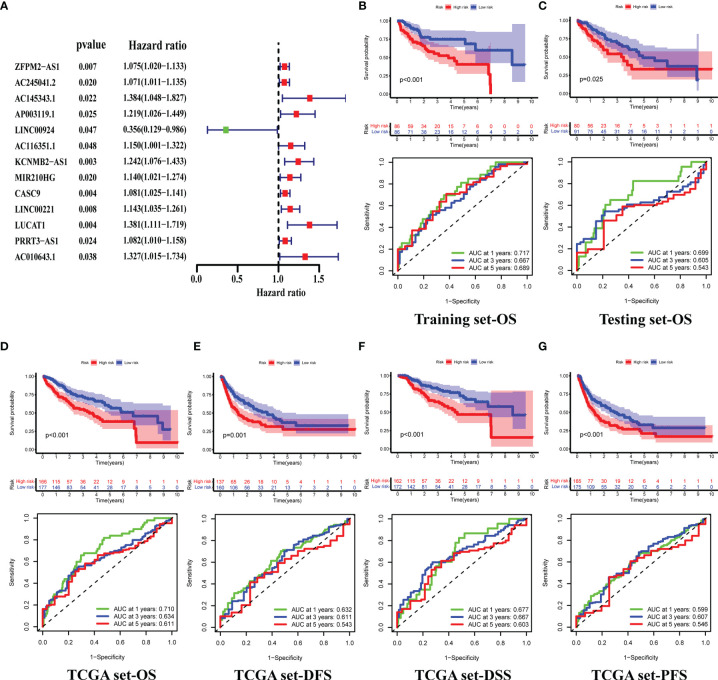
Construction of GILncSig and Validation of its Prognostic Prediction Performance. **(A)** Prognosis-related GIlncRNAs from the training set, screened using univariate Cox regression analysis. **(B–D)** Differences in the overall survival(OS) of HCC patients between the high- and low-risk groups, and 1-,3-,5-year ROC curve of risk scores, in the training **(B)**, testing **(C)**, and TCGA sets **(D)**. **(E–G)** Differences in the Disease-free survival (DFS) **(E)**, Disease-specific survival (DSS) **(F)** and Progression-Free Survival (PFS) **(G)** of HCC patients between the high- and low-risk groups, and 1-,3-,5-year ROC curve of risk scores, in the TCGA set.

A total of 343 patients were randomly divided into a training set (n = 172) and a test set (n = 171) in a 1:1 ratio. We screened 13 prognostic-related GILncRNAs from the training set using univariate Cox regression analysis (**Figure 3A**). Then, we performed multivariate Cox regression analysis on the above 13 GILncRNAs, obtained the correlation coefficient of five GILncRNA, and finally established a GILncSig (**Table 1**). This GILncSig consists of five lncRNAs, including AC245041.2, AP003119.1, MIR210HG, LINC00221, and LUCAT1. The formula of the signature is the following: risk score = expAC245041.2 × 0.086 + expAP003119.1 × 0.173 + expMIR210HG × 0.098 + expLINC00221 × 0.112 + expLUCAT1 × 0.219.

**Table 1:** The lncRNA composition of the GILncSig.

GILncSig	Coefficient	HR	95％CI (low)	95％CI (high)
AC245041.2	0.086	1.090	1.027	1.158
AP003119.1	0.173	1.189	0.971	1.456
MIR210HG	0.098	1.103	0.975	1.248
LINC00221	0.112	1.118	1.001	1.249
LUCAT1	0.219	1.245	0.956	1.622

HR, hazard ratio; CI, confidence interval.

### Evaluating and Validating the GILncSig Based on a TCGA Cohort

Before checking the signature, we found that the two groups of patients were comparable by comparing the clinical information in the training and testing sets (all *p >*0.05, **Table 2**).

**Table 2:** Comparison of clinical data between the training set and testing set.

Covariates	Type	Training set (n=172)	Testing set (n=171)	*p*
**Age (%)**	<=65	61.05	64.91	0.53
	>65	38.95	35.09	
	Unknown	0.00	0.00	
**Gender (%)**	Female	31.98	32.16	1.00
	Male	68.02	67.84	
**Grade (%)**	G1-3	93.6	96.49	0.75
	G4	4.07	2.92	
	Unknown	2.33	0.58	
**Stage (%)**	Stage I-II	68.02	70.76	0.51
	Stage III-IV	26.16	22.22	
	Unknown	5.81	7.02	
**T (%)**	T1-2	73.26	73.68	0.95
	T3-4	26.16	25.15	
	Unknown	0.58	1.17	
**M (%)**	M0	76.16	66.67	0.91
	M1	0.58	1.17	
	Unknown	23.26	32.16	
**N (%)**	N0	72.67	66.67	1.00
	N1	1.16	0.58	
	Unknown	26.16	32.75	

Next, we tested GILncSig for predictive performance. By plotting the survival curves and ROC curves we found that in the training set, the overall survival(OS) of patients in the high-risk group was significantly lower than that of patients in the low-risk group (median OS = 3.8 versus 8.6 years, respectively, *p* < 0.001), and the AUCs for predicting patient OS at 1, 3 and 5 years were 0.717, 0.667 and 0.689 (**Figure 3B**) respectively. This was also validated in the testing and TCGA sets (**Figures 3C, D**). In addition, we also plotted the survival and ROC curves of disease-free survival (DFS), disease-specific survival (DSS), and progression-free survival (PFS) of patients in the TCGA set, respectively. The results all showed that the high-risk group had a worse prognosis than the low-risk group, and GILncSig has a good prediction performance (all *p <*0.05, all AUCs >0.5, **Figures 3E–G**).

**Figure 4:**
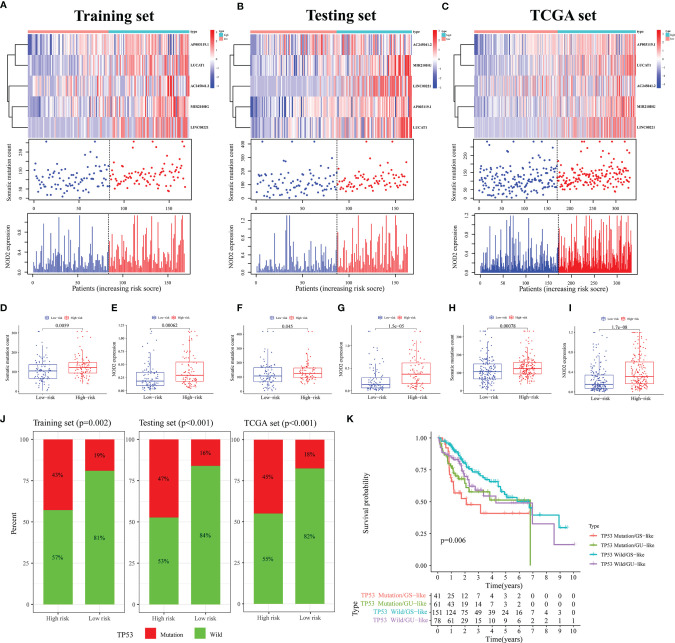
Analysis of Mutation Correlation. **(A–C)** Risk curve consisting of a heat map, mutation point plot, and gene expression plot in the training **(A)**, testing **(B)**, and TCGA sets **(C)**. **(D, F, H)** Differences in the number of somatic mutations between the high- and low-risk groups in the training **(D)**, testing **(F)**, and TCGA sets **(H)**. **(E, G, I)** Differences in the *NOD2* expression level between the high- and low-risk groups in the training **(E)**, testing **(G)**, and TCGA sets **(I)**. **(J)** Comparison of the proportion of the TP53 mutation status in the high- and low-risk groups in the training, testing, and TCGA sets. **(K)** Results of combined survival analysis of TP53 in the different gene states and different clusters.

Furthermore, mutation correlation analysis of the signature was conducted. By drawing a heat map, we found that the GILncSig expression value was upregulated in the high-risk group of the three sets (**Figures 4A–C**). In the mutation point plots and gene expression plots, the number of somatic mutations and the *NOD2* expression level showed an increasing trend in the three sets as the risk value increased (**Figures 4A–C**). Through further validation, we found that the number of mutations was significantly elevated in the high-risk groups in the three sets compared with the low-risk groups (all *p* < 0.05, **Figures 4D, F, H**). Similarly, in these groups, *NOD2* gene expression levels were higher in the high-risk groups (**Figures 4E, G, I**). In addition, LncRNA has a wild and mutation state; thus, we used the signature to verify the proportion of the single-gene mutation states. In this study, TP53 (39, 40) with a high mutation frequency was selected as the validation object. In the training set, we found that the proportion of TP53 mutation status was significantly higher in the high-risk group compared with the low-risk group(p = 0.002, **Figure 4J**). This result was verified in the testing and TCGA sets (all *p* < 0.001, **Figure 4J**). Considering that the different clusters (Gs-like and GU-like) of patients might have different survival rates under different statuses, further analysis was carried out by plotting survival curves. As shown in **Figure 4K**, for the GU group, there was little difference in survival between TP53 mutant and wild-type patients. In the GS group, we found that the prognosis of patients with TP53 mutation was significantly worse than that of patients in the TP53 wild-type group(*p* = 0.006). Taken together, these results suggest that our signature can be a good predictor of the frequency of somatic mutations and the prognosis of patients with HCC.

**Figure 5:**
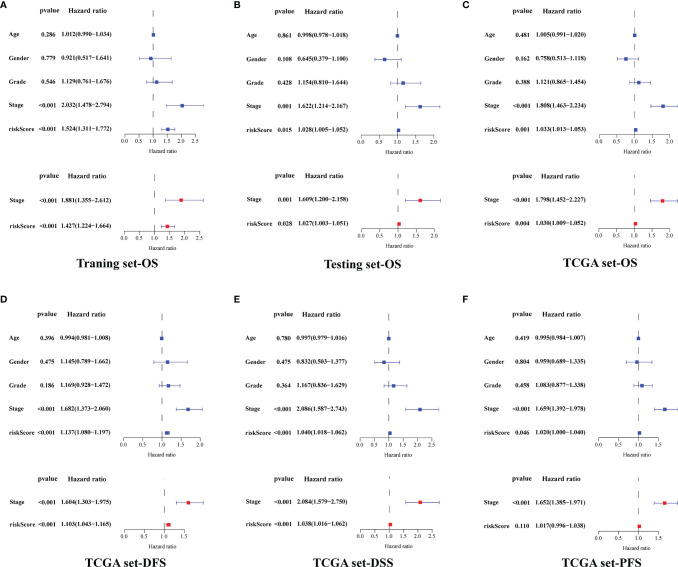
Independent Prognostic Analysis of GILncSig. The Cox regression analysis of the risk score in the training set-OS **(A)**, testing set-OS **(B)**, TCGA set-OS **(C)**, TCGA set-DFS **(D)**, TCGA set-DSS **(E)** and TCGA set-PFS **(F)**. Among them, blue represents univariate Cox regression analysis, and red represents multivariate Cox regression analysis.

Then, we performed an independent prognostic analysis of GILncSig. Univariate and multivariate Cox regression analysis showed that risk score and tumor stage were independent predictors of OS, DSS, and DFS (**Figures 5A–F**).

**Figure 6:**
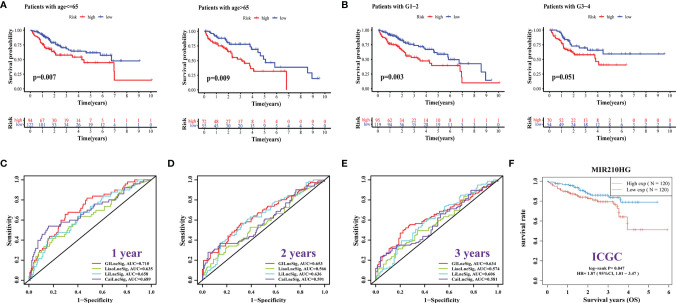
Clinical Stratification Analysis, Performance Comparison and ICGC Cohort Validation of GILncSig. **(A, B)** Comparison of the OS between the high- and low-risk groups of patients who are <= 65 or > 65 years **(A)**, with a grade of G1-2 or G3-4 **(B)**. **(C–E)** Comparison of the area under ROC curve of the 1- **(C)**, 2- **(D)**, and 3-year **(E)** OS between GILncSig of this study and other prognostic signatures in the HCC patients. **(F)** Validation of the relationship between the expression of MIR210HG in GILncSig and OS in the ICGC database.

At last, to understand the applicability of the GILncSig, we performed a clinical stratification analysis of clinical factors including age and tumor grade. We observed that for patients aged <= 65 or > 65 years with HCC, the survival rate was significantly higher in the low-risk group than in the high-risk group (**Figure 6A**, all *p* < 0.05). Similarly, for patients with tumor grades 1-2 (*p* = 0.003) or 3-4 (*p* = 0.051), the survival was higher in the low-risk group than in the high-risk group (**Figure 6B**). This suggests that GILncSig may be generally applicable to patients with HCC.

### Performance Comparison of GILncSig and Verification of ICGC Cohort

To more intuitively validate the GILncSig, we compared it with some existing prognostic models for patients with HCC. The first one was the 4-methylated differentially expressed lncRNAs (4-MDELs) model (AC025016.1, LINC01164, LINC01183, LINC01269) established by Liao (41). The second was the 12-lncRNA model constructed by Li (42) (LINC01018, FAM230B, RP11‐290F5.1, APCDD1L‐AS1, FAM99A, LINC01370, AC002116.8, GRM8, RP11‐30J20.1, RP11‐136K7.2, WDR86AS1, and LINC00671). The last was an immune-related LncRNA model created by Cai (43) (FAM120AOS, AL445524.1, AC073257.2, LINC00513, and STK3). The results showed that the AUC values of the ROC of our signature for the 1-, 2-, and 3-year OS were 0.710, 0.653, and 0.634, respectively, which were significantly higher than those of the other three models (**Figures 6C–E**). These results confirmed that our signature was superior to some existing models in predicting the survival rates of patients with HCC. In addition, we confirmed in the ICGC cohort that high expression of the MIR210HG gene in GILncSig could predict poor prognosis in HCC patients (**Figure 6F**, *p =* 0.047).

### LUCAT1 Expression Was Associated With Poor Prognosis in HCC

**Figure 7:**
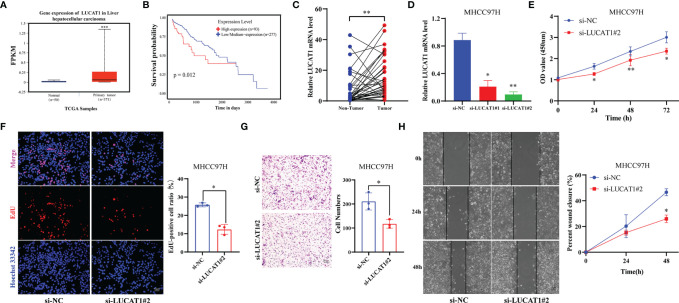
Adverse Effects of LUCTA1 on HCC *in vitro.* **(A)** ENCORI server analyzed the expression of the LUCAT1 gene in HCC. **(B)** Kaplan–Meier curve of the expression level of LUCAT1 on HCC patients using ENCORI. **(C)** qRT-PCR analysis of LUCAT1 mRNA levels in HCC tissues and corresponding adjacent tissues (n=37). **(D)** The efficiency of knockdown of LUCAT1 expression in MHCC97H cells was verified by qRT-PCR. **(E)** After LUCAT1 silencing, the cell viability of MHCC97H was significantly inhibited by the CCK- 8 assay. **(F)** Compared with the control group, the proliferation rate of MHCC97H cells was significantly inhibited after LUCAT1 silencing by EdU staining. **(G)** Transwell experiments showed that the migratory ability of MHCC97H was inhibited after LCUAT1 silencing. **(H)** Wound healing array showed that LUCAT1-downregulated MHCC97H cells exhibited significantly delayed wound healing compared with controls. Scale bar: 50μm, *p <0.05, **p <0.01, ***p <0.001.

In GILncSig, LUCAT1 was the most important lncRNA for predicting poor prognosis based on regression coefficients. Therefore, we further evaluated the function of LUCAT1 in HCC. First, expression differential and survival analyses were performed using the ENCORI Online Tools (https://starbase.sysu.edu.cn). It was observed that LUCAT1 was significantly highly expressed in HCC (**Figure 7A**, *p* < 0.001). Patients with HCC in the low LUCAT1 group had a better prognosis compared to the high LUCAT1 group (**Figure 7B**, *p* = 0.012). The qRT-PCR results showed that the mRNA of LUCAT1 gene was highly expressed in HCC tissues compared to the corresponding paraneoplastic tissues (**Figure 7C**, n=37, *p* = 0.002). Further, to analyze the role of LUCAT1, we transfected si-LUCAT1 into MHCC97H cells, the knockdown effect was shown in **Figure 7D**, and then we used the best knockdown effect si-LUCAT1#2 for the next experiment. To assess the effect of LUCAT1 on proliferation in HCC, we used CCK-8 and EdU staining assays in MHCC97H with/without LUCAT1 knockdown. After interfering with LUCAT1 expression in MHC97H cells, the cell proliferation rate in the si-LUCAT1 group was significantly lower than that in the si-NC group (**Figures 7E, F**). The effect of inhibiting LUCAT1 expression on HCC cell migration was further analyzed. Transwell assays and wound-healing experiments together showed that the migratory ability of MHCC97H cells was significantly reduced upon inhibition of LUCAT1 expression (**Figures 7G, H**). In summary, *in vitro* experimental data suggest that high expression of LUCAT1 is closely related to poor prognosis in patients with HCC.

### Exploring the Possible Functions and Pathways of GILncSig

**Figure 8:**
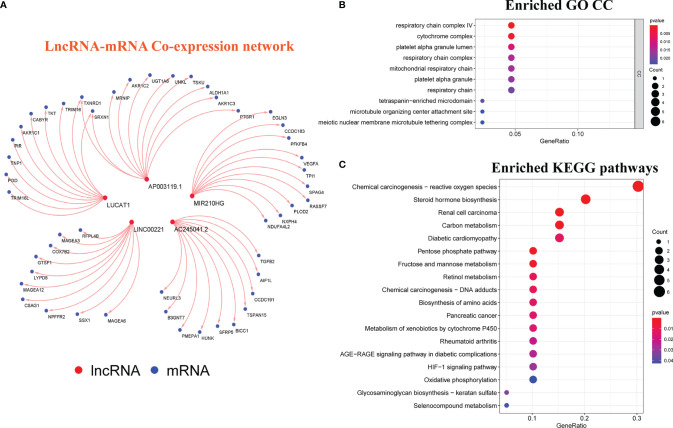
Exploration of the Possible Functions and Pathways of GILncSig. **(A)** LncRNA-mRNA co-expression network diagram. **(B)** Analysis of the Cellular Component (CC) terms of Gene Ontology (GO) enrichment demonstrated the possible function of the genome instability-related lncRNA signature (GILncSig). **(C)** Kyoto Encyclopedia of Genes and Genomes (KEGG) pathway enrichment analysis revealed the possible pathways associated with genomic instability (GI).

To understand the underlying mechanisms *via* which our signature affected GI, we first mapped the lncRNA-mRNA co-expression network (**Figure 8A**). Then, the possible functions and pathways were discovered using GO and KEGG enrichment analyses. The cellular component (CC) of GO enrichment analysis revealed that the GILncSig-related mRNA was closely connected with the occurrence and development of GI, including the respiratory chain, respiratory chain complex, microtubule-organizing center, attachment site, and meiotic nuclear membrane microtubule tethering complex, among others (**Figure 8B**). Through KEGG pathway analysis, we found that chemical carcinogens-reactive oxygen species, oxidative phosphorylation, HIF-1 signaling pathway, and chemical carcinogenesis, DNA adducts, pentose phosphate pathway, Fructose and mannose metabolism, and other significantly enriched pathways were also significantly associated with GI related. (**Figure 8C**). These results indicated that our GILncSig was involved in GI and its altered expression might destruct the GS of cells and increase GI by interfering with DNA meiosis, microtubule tissue center attachment, and DNA adsorption to affect the normal gene damage repair pathways.

### Association of GILncSig With Immune Cell Infiltration and Immune Checkpoints

**Figure 9:**
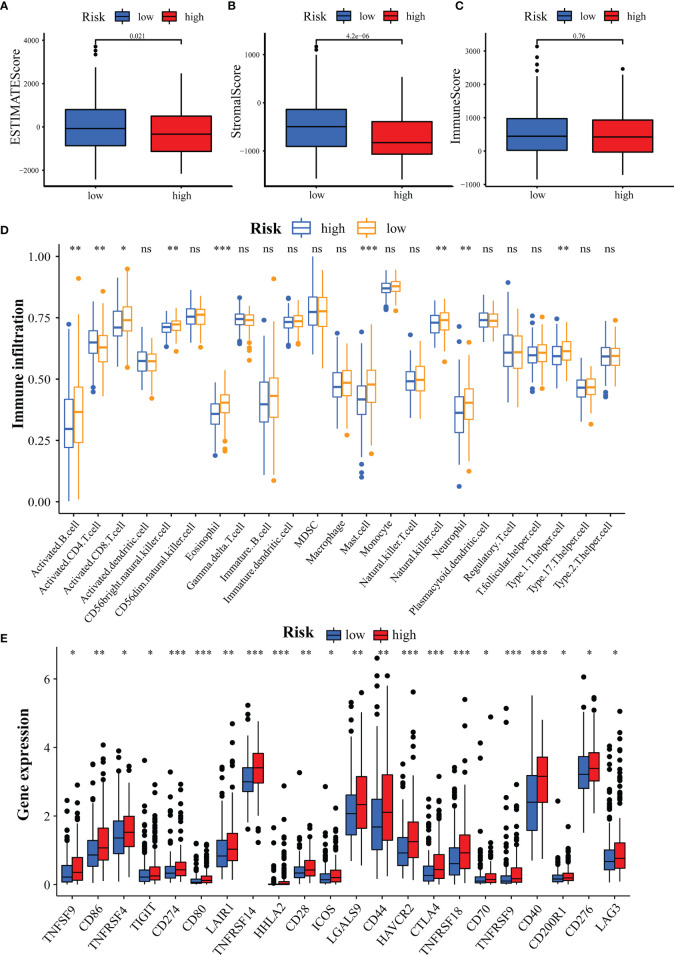
Assessment of Tumor Microenvironment, Immune Cell Infiltration and Immune Checkpoint Genes in Different Groups. **(A-C)** Comparison of ESTIMATE score **(A)**, stromal score **(B)**, and immune score **(C)** between the high- and low-risk groups. **(D)** Differences in the infiltration of immune cells between the high- and low-risk groups. **(E)** Differential expression of immune checkpoint genes between the high- and low-risk groups. *p<0.05, **p<0.01, ***p<0.001; ns, not statistically different.

Next, we analyzed the association of GILncSig with the immune status of patients in the TCGA-HCC cohort. High tumor purity has been reported to be associated with immune cell infiltration and cancer development and prognosis (44). Hence, we predicted the stromal score, immune score, and ESTIMATE score in the high- and low-risk groups by the ESTIMATE algorithm. The results demonstrated no difference in immune scores between groups (**Figure 9C**, *p* = 0.76), while there were significant differences in stromal and ESTIMATE scores (**Figures 9A, B**, all *p* < 0.05). Then, we analyzed the correlation between GILncSig and 23 immune cells using the ssGSEA algorithm. The results showed that activated B cells, activated CD8 T cells, CD56 natural killer cells, eosinophils, mast cells, natural killer cells, neutrophils, and type 1 T helper cells were more predominant in the low-risk group, while activated CD4 T cells were more predominant in the high-risk group (**Figure 9D**). It has also been reported that an extremely promising approach to achieve anti-cancer immunity is to block the immune checkpoint pathway (45). Therefore, we analyzed the differences in checkpoint gene expression between the high- and low-risk groups. The result was shown in **Figure 9E**, which indicated that the expression of all checkpoint genes was statistically significant between the two groups, especially for CD274(PD-L1), CD80, TNFRSF14, HHLA2, HAVCR2, CTLA4, TNFRDF9, TNFRSF9, and CD40 (*p* < 0.001). These results suggest that GILncSig can be used to assess the tumor immune microenvironment and the expression of immune checkpoint genes in patients with HCC.

### GILncSig Predicts Efficacy of Chemotherapy and Immunotherapy Response

**Figure 10:**
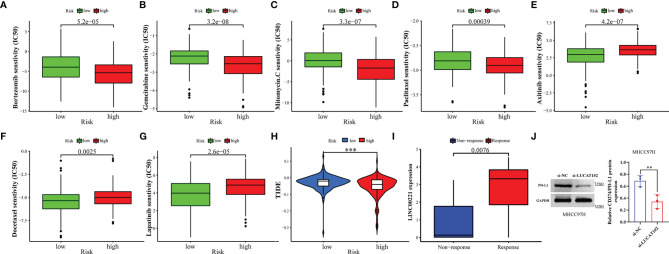
GILncSig predicts chemotherapy and immunotherapy response. **(A–G)** The signature showed high-risk scores were associated with a lower IC50 for chemotherapeutics such as **(A)** bortezomib, **(B)** gemcitabine, **(C)** imatinib, and **(D)** paclitaxel, whereas they were related to a higher IC50 for **(E)** axitinib, **(F)** docetaxel, and **(G)** lapatinib. **(H)** Differences in TIDE score between high- and low-risk groups. **(I)** Different expression of LINC00221 predicts response to anti-PD-1 immune checkpoint inhibition therapy in HCC patients. **(J)** Effects of with or without inhibition of LUCAT1 expression on PD-L1 protein expression levels by western blotting. **p<0.01,***p<0.001.

To assess the predictive effect of GILncSig on drug therapy for liver cancer, we analyzed the relationship between high- and low-risk groups and the efficacy of commonly used chemotherapeutic agents. Our study showed that the low-risk group was significantly associated with a higher IC50 for chemotherapeutic agents such as bortezomib, gemcitabine, mitomycin, and paclitaxel (**Figures 10A–D**, all *p*<0.001). In contrast, the high-risk group was more sensitive to treatment with axitinib, docetaxel, and lapatinib (**Figures 10E–G**, all *p*<0.01). In immunotherapy, we assessed the effectiveness of immune checkpoint (PD-1 and CTLA-4) inhibitors by TIDE scores in the high- and low-risk groups. TIDE scores were significantly lower in high-risk patients compared to low-risk patients (**Figure 10H**, *p* < 0.001), suggesting that patients in the high-risk group may have better efficacy when receiving immunosuppressive drugs. With the immunotherapy GSE78220 cohort, we also validated that patients with high LINC00221 gene expression in GILncSig responded better to immunosuppressive therapy (**Figure 10I**, *p*=0.0076). Interestingly, we found that inhibiting the expression of LUCAT1 reduced the protein expression of PD-L1 by western blotting (**Figure 10J**, *p* = 0.0024).

## Discussion

Liver cancer is a kind of malignant tumor with the fastest rising incidence and a very low survival rate (46), and its main pathological type is HCC (2). Except for patients with HCC who are diagnosed early and receive potentially curative treatment (surgical resection and liver transplantation), the overall prognosis of patients is disheartening (47). Thus, exploring valuable signatures is important in assessing the prognosis and treatment of patients with HCC. LncRNA is a class of non-coding protein RNA molecules (18) that function at various levels of gene regulation by forming networks with DNA, protein, and RNA molecules (48). Thus, researchers have begun to use LncRNA as a biomarker to predict the prognosis of patients with HCC (49–56). In past studies, these lncRNAs were usually related to autophagy and immunity. However, the lncRNAs associated with GI, which is regarded as an important driver of cancer, are rarely reported. This makes it urgent to establish a GILncSig for HCC patients to help predict prognosis. Accordingly, in this study we screened 88 GILncRNAs and validated the relationship between these lncRNAs and GI after clustering. Then, we obtained prognosis-related GILncRNAs by univariate regression analysis and correlation coefficients of these lncRNAs by multivariate regression analysis to establish GILncSig. Next, we evaluated GILncSig by various aspects such as survival analysis, mutation correlation analysis, independent prognosis analysis, model comparison, *in vitro* experiments among others. Taken together, these results indicated that GILncSig could be used as a promising prognostic indicator.

Our GILncSig included MIR210HG, LINC00221, LUCAT1, AC245041.2, and AP003119.1, all of which were prognostic risk genes for patients with HCC. The effects of LINC00221, LUCAT1, and MIR210HG on patients with HCC have been mentioned in previous studies (57–61). In addition, LINC00221 could predict OS in patients with gastric cancer (62) and modulated cisplatin treatment resistance in non-small cell lung cancer (63). In this study, we found for the first time with the GSE78220 cohort that high expression of LINC00221 might be associated with a better response of patients to immunosuppressive therapy. LUCAT1 also plays a role in many cancers. For instance, the LUCAT1/miR-5582-3p/TCF7L2 axis adjusts breast cancer stemness *via* the Wnt/β-catenin pathway (64), and LUCAT1 promotes the occurrence and development of esophageal squamous cell carcinoma, colorectal cancer, and gastric cancer by controlling the ubiquitination and stability of DNA methyltransferase 1 (65). Based on regression coefficients, LUCAT1 was seen as the most important GIlncRNA for risk factors and prognostic prediction. we also demonstrated through online database analysis that LUCAT1 was associated with poor prognosis in HCC patients. *In vitro*, interference with LUCAT1 expression inhibited HCC proliferation and migration, similar to previous findings (60). Interestingly, we found for the first time that inhibition of LUCAT1 led to downregulation of CD274(PD-L1) protein expression, which could provide new insights for immunotherapy. MIR210HG was found to be a poor prognostic factor for patients with pancreatic, endometrial, glioma, osteosarcoma, and colorectal adenocarcinoma (66–70). We also validated that high MIR210HG expression was associated with poor prognosis in patients with HCC by the ICGC cohort. AC245041.2 could be considered as a poor prognostic factor for patients with pancreatic cancer (71–73) and head and neck squamous cell carcinoma (74). In addition, AP003119.1, which has not been mentioned in previous studies, was used as a biomarker for HCC in our study for the first time. However, further research in the future is needed to understand the deeper mechanisms.

Subsequently, we discovered the functions and signaling pathways of GILncSig related to GI through GSEA. For example, reactive oxygen species (ROS) produced by the action of NADPH oxidase (75), can cause DNA double-strand breaks (DSB) and repair changes, leading to GI (76). Moreover, ROS are involved in inducing DNA damage, DSB sensing, DDR signal transduction, cell cycle progression, p53 transcriptional response, apoptosis, and DNA repair, among others (77). *SDHD* mutation (78) and *SDHC* mutation (79)could also lead to ROS formation. The respiratory chain is composed of a series of electron carriers and can transfer electrons from NADH or FADH2 to oxygen. When it mutates or its function changes, it probably leads to nuclear GI (80). The pathogenesis of respiratory chain complex IV deficiency may also be associated with GI (81). The microtubule-organizing center (MTOC) is a structure that forms microtubules in eukaryotic cells and organizes the formation of the spindle during meiosis or mitosis. The centrosome is the main MTOC (82). Some studies have indicated that GI in HCC can be induced by centrosome aberrations related to a p53 mutation or occurring during a stable HBx transfection (83, 84). DNA adducts formed by the biotransformation of cytochrome p450 enzyme can also cause GI (85). For example, both AFB1-FAPY adducts and AFB1-N7-Gua adducts lead to a G to T transposition (86, 87). The pentose phosphate pathway and HIF-1 pathway are also involved in GI regulation. For instance, ATM activates the pentose phosphate pathway by promoting anti-oxidant defense and DNA repair (88); the p53-TIGAR axis suppresses glycolysis in FA HSCs, leading to an enhanced pentose phosphate pathway and cellular anti-oxidant function and, consequently, to reduced DNA damage and GI (89); the p38α stress kinase suppresses aneuploidy tolerance by inhibiting Hif-1α (90). In addition, fructose metabolism, retinol metabolism, and rheumatoid arthritis are all related to GI (91–95). These findings indicate that our characteristics are intimately linked to the occurrence and development of GI.

HCC is an inflammation-associated malignancy that contains multiple immune cell subtypes that form a complex immune tolerance microenvironment that promotes HCC (96). In this exercise, we found that the high-risk group had lower stromal cell infiltration. This suggests to some extent that GILncSig can predict the composition of TME. In addition, we analyzed the differences in the abundance of 23 immune cell types between the high- and low-risk groups. Activated B cells, activated CD8 T cells, CD56 natural killer cells, and natural killer cells were reported to be tumor antagonistic immune cells (97). Here, we found more levels of tumor antagonistic immune cells in the low-risk group compared to the high-risk group. This suggests that GILncSig can be used to assess the tumor immune microenvironment in HCC patients. As immune checkpoint therapies were incorporated into HCC therapy, their combination with molecularly targeted therapies is emerging as a tool to enhance the immune response. We found that patients with high-risk scores had higher expression of immune checkpoint genes, suggesting that our signature might be used to evaluate the expression of immune checkpoint genes. Finally, we estimated susceptibility to commonly used chemotherapeutics and immunotherapy responses in high- and low-risk groups based on the pRRophetic algorithm and the TIDE program. Our results demonstrated the potential predictive value of GILncSig for chemosensitivity and immunotherapy efficacy. In addition, we performed external validation of some genes in GILncSIg by immunotherapy GSE78220 cohort and *in vitro* experiments. Take together, GILncSig will help to select patients suitable for chemotherapy, targeted drugs, and immunotherapy, and provide new ideas for developing treatment strategies to improve the disease prognosis of HCC patients.

However, our study also has limitations. This study was partially validated in *in vitro* experiments and in the ICGC cohort, lacking full validation from external datasets. In the future, we will continue to collect sufficient samples to assess the value of this signature in combination with immunotherapy and to verify whether the benefit of immunotherapy differs between high- and low-risk groups.

In sum, we have exploited relevant lncRNAs from a GI perspective to develop a new signature in patients with HCC. This will help clinicians evaluate the overall prognosis of patients and provide new insights for clinical decision-making and potential therapeutic strategies.

## Data Availability Statement

Publicly available datasets were analyzed in this study. The data can be found here: https://portal.gdc.cancer.gov/, The Cancer Genome Atlas (TCGA); https://dcc.icgc.org/releases/current/Projects, The International Cancer Genomics Consortium (ICGC, LIRI-JP); https://www.ncbi.nlm.nih.gov/geo/query/acc.cgi?acc=gse78220, GSE77820. Therefore, this study was exempted from the approval of the local ethics committee. The current study follows TCGA, ICGC, and GEO data access policies and publication guidelines.

## Ethics Statement

The studies involving human participants were reviewed and approved by The Second Affiliated Hospital of Nanchang University Medical Research Ethics Committee Review Documents. The patients/participants provided their written informed consent to participate in this study.

## Author Contributions

JZ and QH conducted the formal analysis and wrote the original draft. RY performed the project administration. SL and XP conducted the experiments. JX, TF, WH, and ZC participated in software analysis. KL, YJ, and MW conducted data curation. JZ, QH, and RY contributed to writing, reviewing, and editing the article. RY provided funding acquisition. All authors read and approved the final submitted manuscript.

## Funding

This study was supported by the National Natural Science Foundation of China (Nos.81960436 and 81560396) the Project of the Jiangxi Provincial Department of Science and Technology (Nos. 20202BBGL73037, 20192BCB23023 and 20181BBG70011), Double Thousand Talents Project of Jiangxi Province (No.jxsq2019201100).

## Conflict of Interest

The authors declare that the research was conducted in the absence of any commercial or financial relationships that could be construed as a potential conflict of interest.

## Publisher’s Note

All claims expressed in this article are solely those of the authors and do not necessarily represent those of their affiliated organizations, or those of the publisher, the editors and the reviewers. Any product that may be evaluated in this article, or claim that may be made by its manufacturer, is not guaranteed or endorsed by the publisher.
