# Supplementary material for: Construction of a Novel LncRNA Signature Related to Genomic Instability to Predict the Prognosis and Immune Activity of Patients With Hepatocellular Carcinoma
Source: Front Immunol. 2022 Apr 8;13:856186. doi: 10.3389/fimmu.2022.856186 (PMC9037030; doi:10.3389/fimmu.2022.856186)
Supplement: Supplementary file 1 [file Table_1.docx]

| name | sequences |
| --- | --- |
| **Primers for real-time PCR:** |  |
| LUCAT1 sense: | 5'-TGATGCTGAGTTTTGGAGTATGC-3′ |
| LUCAT1 antisense: | 5'-GGGAAGGGAGTGAAGTAAATGTTA-3′ |
| GAPDH sense: | 5'-AGAAGGCTGGGGCTCATTTG-3′ |
| GAPDH antisense: | 5'-AGGGGCCATCCACAGTCTTC-3′ |
| **The target sites of shRNA:** |  |
| si-LUCAT1#1 sense： | 5'-CCAGACCUCCAGAAACCAUTT-3′ |
| si-LUCAT1#1 antisense： | 5'-AUGGUUUCUGGAGGUCUGGTT-3′ |
| si-LUCAT1#2 sense： | 5'-CCAACUUGCUGUUUGCUAUTT-3′ |
| si-LUCAT1#2 antisense： | 5'-AUAGCAAACAGCAAGUUGGTT-3′ |
| si-NC sense： | 5'-UUCUCCGAACGUGUCACGUTT-3′ |
| si-NC antisense： | 5'-ACGUGACACGUUCGGAGAATT-3′ |

**Supplementary Table1. Primers and siRNA target sequences.**
