# Supplementary material for: Construction of a Novel LncRNA Signature Related to Genomic Instability to Predict the Prognosis and Immune Activity of Patients With Hepatocellular Carcinoma
Source: Front Immunol. 2022 Apr 8;13:856186. doi: 10.3389/fimmu.2022.856186 (PMC9037030; doi:10.3389/fimmu.2022.856186)
Supplement: Supplementary file 2 [file Table_2.docx]

**Supplementary Table2. Genomic instability-related LncRNAs (GILncRNAs) in patients with HCC**

| **lncRNA** | **conMean** | **treatMean** | **logFC** | ***p*** | **FDR** |
| --- | --- | --- | --- | --- | --- |
| BX640514.2 | 0.771251 | 0.150899 | -2.35361 | 0.001223 | 0.009053 |
| AC007406.2 | 0.488158 | 1.789562 | 1.874187 | 0.000213 | 0.003024 |
| AC004540.1 | 0.792379 | 0.145279 | -2.44737 | 0.00176 | 0.011877 |
| AC096996.2 | 0.247048 | 0.631955 | 1.355032 | 0.000156 | 0.002493 |
| AP003174.1 | 0.298448 | 0.615395 | 1.044031 | 0.008289 | 0.033482 |
| AL390719.2 | 0.660548 | 0.243263 | -1.44115 | 0.000275 | 0.003572 |
| ZFPM2-AS1 | 0.552893 | 2.048777 | 1.889692 | 3.90E-05 | 0.000857 |
| AC245041.2 | 1.195797 | 0.147979 | -3.01451 | 0.00738 | 0.031394 |
| AL035661.1 | 4.443602 | 2.072519 | -1.10034 | 0.000147 | 0.00242 |
| AL162413.1 | 0.857553 | 5.262505 | 2.617452 | 7.14E-06 | 0.000259 |
| LINC01639 | 0.192009 | 0.618975 | 1.688703 | 0.008106 | 0.032956 |
| AP003119.2 | 0.983604 | 2.098563 | 1.093253 | 2.40E-07 | 3.52E-05 |
| LINC02163 | 0.280085 | 1.404049 | 2.325659 | 4.42E-11 | 5.91E-08 |
| LINC02298 | 1.089846 | 0.480602 | -1.18121 | 0.000259 | 0.003434 |
| AL133355.1 | 0.650488 | 0.315158 | -1.04545 | 4.48E-06 | 0.000189 |
| LINC01287 | 0.672504 | 3.55188 | 2.400969 | 0.00341 | 0.018357 |
| AC104083.1 | 2.778417 | 1.020951 | -1.44435 | 8.14E-09 | 2.77E-06 |
| AP001271.1 | 0.633489 | 0.183117 | -1.79055 | 0.005927 | 0.026714 |
| AC145343.1 | 0.430185 | 0.967943 | 1.169966 | 4.67E-06 | 0.000189 |
| LINC02404 | 0.321351 | 0.932819 | 1.537448 | 1.66E-05 | 0.000461 |
| Z82246.1 | 0.814875 | 1.753288 | 1.105412 | 0.004822 | 0.023101 |
| HHIP-AS1 | 0.669291 | 0.220655 | -1.60084 | 1.09E-05 | 0.000352 |
| AC015922.2 | 1.060114 | 0.268951 | -1.97881 | 8.67E-11 | 5.91E-08 |
| AP003119.1 | 0.499565 | 1.107404 | 1.148437 | 0.000537 | 0.005152 |
| LINC00924 | 0.71606 | 0.284644 | -1.33092 | 2.50E-08 | 4.87E-06 |
| AC010205.1 | 0.317463 | 0.649002 | 1.031635 | 0.002928 | 0.016969 |
| AL691420.1 | 0.323082 | 1.475888 | 2.191611 | 4.36E-05 | 0.000942 |
| AL731684.1 | 0.378848 | 0.844304 | 1.156144 | 0.004402 | 0.02186 |
| LINC01980 | 0.406528 | 2.320032 | 2.512717 | 2.26E-08 | 4.87E-06 |
| AP000593.3 | 0.720826 | 2.033989 | 1.496588 | 0.01441 | 0.049845 |
| AC254562.2 | 0.467288 | 1.005202 | 1.105101 | 0.004937 | 0.023513 |
| AC116351.1 | 0.739925 | 0.154355 | -2.26112 | 0.000106 | 0.002059 |
| AC090809.1 | 0.13774 | 0.739759 | 2.425111 | 1.93E-06 | 0.000119 |
| LINC01151 | 1.633527 | 3.423477 | 1.067472 | 1.63E-06 | 0.000107 |
| KCNMB2-AS1 | 0.252816 | 1.043587 | 2.045394 | 4.31E-07 | 4.89E-05 |
| LINC01480 | 0.810571 | 0.168488 | -2.26629 | 0.012623 | 0.045242 |
| LINC01857 | 0.837464 | 0.305889 | -1.45302 | 0.001474 | 0.010349 |
| LINC02476 | 0.179503 | 0.665703 | 1.890874 | 2.06E-06 | 0.000122 |
| AP000424.2 | 0.957522 | 0.369622 | -1.37326 | 0.000349 | 0.004057 |
| PRR26 | 1.214641 | 0.524063 | -1.21272 | 0.000477 | 0.004811 |
| MIR210HG | 0.981768 | 2.074321 | 1.079186 | 0.001526 | 0.010661 |
| AC016735.1 | 4.551152 | 0.847096 | -2.42563 | 0.000202 | 0.002957 |
| LINC02241 | 0.318498 | 1.102626 | 1.791588 | 1.58E-06 | 0.000107 |
| UCA1 | 3.690452 | 0.100196 | -5.2029 | 0.000201 | 0.002957 |
| LINC02506 | 0.920345 | 2.689253 | 1.546959 | 0.001003 | 0.007898 |
| CASC9 | 1.040077 | 2.786492 | 1.42176 | 1.01E-06 | 8.59E-05 |
| AC090015.1 | 0.37197 | 0.911059 | 1.29236 | 1.49E-05 | 0.000423 |
| AC004080.2 | 0.28188 | 0.644243 | 1.192526 | 0.001995 | 0.012941 |
| LINC01608 | 0.542104 | 1.195605 | 1.141101 | 1.49E-05 | 0.000423 |
| SPINT1-AS1 | 2.106141 | 0.509828 | -2.04652 | 0.000121 | 0.002287 |
| ST8SIA6-AS1 | 0.681824 | 2.746899 | 2.010333 | 3.00E-09 | 1.36E-06 |
| AC010547.2 | 3.033767 | 0.283119 | -3.42163 | 2.50E-06 | 0.000142 |
| IGF2-AS | 1.010289 | 0.479797 | -1.07427 | 0.00075 | 0.00645 |
| PRKAR1B-AS2 | 2.860171 | 0.453063 | -2.65832 | 0.001217 | 0.009053 |
| LINC00221 | 0.508708 | 1.663049 | 1.708922 | 0.001388 | 0.01 |
| AC008035.1 | 1.264517 | 2.690302 | 1.089182 | 0.000151 | 0.00245 |
| AC026462.3 | 2.183324 | 4.642303 | 1.088314 | 0.00083 | 0.006849 |
| LINC01139 | 0.321127 | 0.904339 | 1.49372 | 9.48E-05 | 0.001872 |
| AP001189.3 | 0.696267 | 0.202943 | -1.77856 | 3.50E-08 | 5.96E-06 |
| AL365226.1 | 0.080289 | 0.814808 | 3.343189 | 0.00617 | 0.027283 |
| AP000757.2 | 1.151282 | 0.483972 | -1.25025 | 4.05E-06 | 0.000184 |
| AL355102.4 | 4.428485 | 1.80271 | -1.29665 | 0.000421 | 0.004476 |
| AC092490.1 | 0.430923 | 0.962402 | 1.159211 | 0.000112 | 0.002145 |
| AC013275.1 | 1.4368 | 0.379109 | -1.92218 | 0.00013 | 0.002323 |
| AC079949.2 | 0.721979 | 1.451686 | 1.007701 | 0.0014 | 0.010033 |
| AC004540.2 | 1.301922 | 0.3517 | -1.88823 | 0.000248 | 0.003375 |
| AL023803.1 | 0.915028 | 2.146995 | 1.23043 | 1.22E-05 | 0.000385 |
| AL109917.1 | 0.27274 | 0.646098 | 1.244228 | 5.47E-07 | 5.48E-05 |
| LUCAT1 | 0.194437 | 0.803617 | 2.047205 | 0.002708 | 0.015895 |
| AC004862.1 | 1.05942 | 2.544708 | 1.264225 | 0.002654 | 0.015646 |
| DCXR-DT | 2.58161 | 5.566518 | 1.108504 | 0.000578 | 0.005391 |
| LINC02315 | 0.297369 | 0.959942 | 1.690694 | 0.000129 | 0.002323 |
| AC016405.3 | 0.563488 | 1.169491 | 1.053425 | 0.000395 | 0.004266 |
| LINC01419 | 2.36381 | 6.488975 | 1.456877 | 4.71E-06 | 0.000189 |
| AL512408.1 | 0.325157 | 0.658141 | 1.017259 | 3.67E-06 | 0.000178 |
| LINC01124 | 1.983094 | 5.039624 | 1.345563 | 6.84E-05 | 0.001455 |
| LINC01474 | 1.154815 | 2.351621 | 1.025994 | 0.001192 | 0.00892 |
| PRRT3-AS1 | 1.051024 | 2.180435 | 1.052821 | 0.000202 | 0.002957 |
| AC015922.3 | 1.213113 | 0.516374 | -1.23223 | 5.63E-07 | 5.48E-05 |
| AC010643.1 | 0.295871 | 0.691884 | 1.225563 | 0.000358 | 0.004057 |
| AL512353.1 | 0.272761 | 0.635043 | 1.219217 | 9.99E-06 | 0.000332 |
| AC002456.1 | 0.657802 | 1.455778 | 1.146065 | 1.24E-05 | 0.000385 |
| Z73429.1 | 0.276905 | 0.60199 | 1.120346 | 0.006015 | 0.026775 |
| MEG3 | 4.419059 | 1.665051 | -1.40817 | 3.12E-06 | 0.000157 |
| AP001972.4 | 0.028711 | 1.494515 | 5.701943 | 0.000902 | 0.007186 |
| AGAP2-AS1 | 2.400164 | 1.05166 | -1.19046 | 1.42E-06 | 0.000107 |
| BX649601.1 | 0.318292 | 0.822349 | 1.369399 | 0.001882 | 0.012506 |
| AP000424.1 | 1.119199 | 0.514444 | -1.12138 | 0.007894 | 0.032537 |

conMean, The mean expression level of lncRNA in the genome stable(GS) group; treatMean, The mean expression level of lncRNA in the genome unstable(GU) group; FDR, adjusted p value, fdr<0.05 means the statistical difference.
